# Evasion of Host Antiviral Innate Immunity by Paramyxovirus Accessory Proteins

**DOI:** 10.3389/fmicb.2021.790191

**Published:** 2022-01-31

**Authors:** Chongyang Wang, Ting Wang, Liuyuan Duan, Hui Chen, Ruochen Hu, Xiangwei Wang, Yanqing Jia, Zhili Chu, Haijin Liu, Xinglong Wang, Shuxia Zhang, Sa Xiao, Juan Wang, Ruyi Dang, Zengqi Yang

**Affiliations:** College of Veterinary Medicine, Northwest A&F University, Xianyang, China

**Keywords:** paramyxoviruses, immune evasion, accessory proteins, IFN, antiviral innate immunity

## Abstract

For efficient replication, viruses have developed multiple strategies to evade host antiviral innate immunity. Paramyxoviruses are a large family of enveloped RNA viruses that comprises diverse human and animal pathogens which jeopardize global public health and the economy. The accessory proteins expressed from the P gene by RNA editing or overlapping open reading frames (ORFs) are major viral immune evasion factors antagonizing type I interferon (IFN-I) production and other antiviral innate immune responses. However, the antagonistic mechanisms against antiviral innate immunity by accessory proteins differ among viruses. Here, we summarize the current understandings of immune evasion mechanisms by paramyxovirus accessory proteins, specifically how accessory proteins directly or indirectly target the adaptors in the antiviral innate immune signaling pathway to facilitate virus replication. Additionally, some cellular responses, which are also involved in viral replication, will be briefly summarized.

## Introduction

Paramyxoviruses represent a large family of RNA viruses that cause vital human and animal diseases. For instance, measles virus (MeV), human parainfluenza virus (HPIV), and mumps virus (MuV) are highly infectious worldwide human pathogens. At the same time, zoonotic viruses such as Hendra virus (HeV) and Nipah virus (NiV) induce significant morbidity and mortality in humans. In addition, Newcastle disease virus (NDV), peste des petits ruminants virus (PPRV), and canine distemper virus (CDV) place a heavy economic burden on the animal farming industry.

**FIGURE 1:**
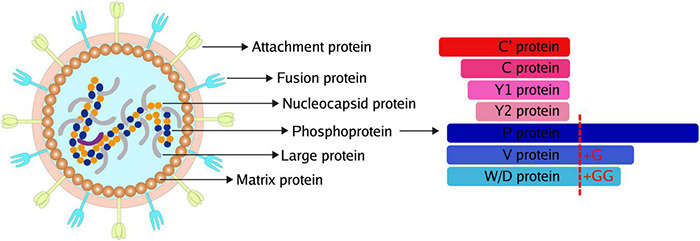
Common Structure and accessory proteins of paramyxoviruses. The common structure of paramyxoviruses **(Left)**. The potential accessory proteins are encoded by paramyxoviruses **(Right)**.

Paramyxoviruses are enveloped viruses with a non-segmented negative-strand RNA genome of 15-19 kb. The genome contains six to ten genes, including the P gene, encoding the phosphoprotein (P) subunit of RNA-dependent RNA polymerase. Except for the phosphoprotein, a panel of accessory proteins is expressed from the P gene by two means: RNA editing for V/W/D proteins and overlapping open reading frames (ORFs) for C proteins (Figure 1). Commonly, insertion of a single G residue produces V protein, while W/D proteins are produced from mRNAs with two inserted G residues. The accessory proteins produced from RNA editing usually share the N-terminal domain with P and have a distinct C-terminal domain. In addition, C protein is expressed using different translation initiation codons. Sendai virus (SeV) produces various C proteins, including C′, C, Y1, and Y2. Meanwhile, henipavirus only express a single species of C protein with 166 amino acids.

Pattern recognition receptors (PRRs) recognize various pathogen-associated molecular patterns (PAMPs) and activate the type I interferon (IFN-I) signaling pathway to inhibit viral replication. Toll-like receptors (TLRs) are well-characterized PRRs. TLRs recognizes nucleic acids derived from viruses, including TLR3, TLR7, TLR8, and TLR9, which subsequently recruit Toll/interleukin-1 receptor (TIR) domain-containing adaptors, including adaptor protein myeloid differentiation factor 88 (MyD88), MyD88 binding protein-like protein (Mal; also known as TIRAP), TIR domain-containing adaptor inducing IFN-β (TRIF), TRIF-related adaptor molecules (TRAM) and Sterile-alpha and Armadillo motif-containing protein (SARM) (Takeuchi and Akira, 2010; Zhu and Zheng, 2020). According to previous studies, paramyxoviruses can be detected by TLR3, TLR7, TLR8, and TLR9 (Melchjorsen et al., 2005; Kitagawa et al., 2011, 2013; Cheng et al., 2014). In response to stimulation with virus-derived double-strand RNA (dsRNA), TLR3 recruits TRIF and promotes TRAF3 (TNF receptor-associated factor 3)-mediated activation of the TBK1 (TANK-binding kinase 1)-IRF3 (IFN regulatory factor 3) axis to induce the IFN and IFN-stimulated genes (ISGs) expression. TLR7, TLR8, and TLR9 signaling promote the induction of IFN-I in a MyD88-dependent manner. Then IRF7 or IRF1 is activated to induce the expression of genes encoding IFN-I in a cell-line-dependent manner (Negishi et al., 2006; Schmitz et al., 2007).

Retinoic acid-inducible gene I (RIG-I)-like receptor (RLR) family, composed of three central members: RIG-I, melanoma differentiation-associated gene 5 (MDA5), and laboratory of genetics and physiology 2 (LGP2), can recognize paramyxovirus RNA and bind to the mitochondrial antiviral signaling (MAVS) protein, thus activating the MAVS-mediated signal transduction to induce IFN-I and proinflammatory cytokines. In addition to TLRs and RLRs, the nucleotide-binding oligomerization domain (NOD)-like receptors (NLRs) are another subfamily of PRRs, which are involved in sensing paramyxoviruses during the host antiviral innate immune responses (Su et al., 2016; Shil et al., 2018; Zhu and Zheng, 2020).

However, to evade host antiviral innate immunity, paramyxoviruses have evolved multiple strategies to facilitate their replication. This review will summarize and update recent findings of the paramyxoviruses accessory proteins (including P, V, W, and C proteins) that attenuate host antiviral innate immune signaling pathways. The canonical PRR-mediated production of IFN-I response and other cellular defense responses, such as stress granule (SG), autophagy, and apoptosis mediated-antiviral innate immunity, will be included.

## Accessory Proteins Inhibit the Pattern Recognition Receptor-Mediated Antiviral Innate Immunity

The PRRs are major components of innate immunity and are capable of recognizing virus infection. TLRs, RLRs, and NLRs play important roles in detecting paramyxoviruses and promoting IFN-I and ISGs. To survive and proliferate in host cells, paramyxoviruses express accessory proteins to counteract the innate antiviral immunity. In this section, the PRR-mediated classical innate immune responses and the downstream IFNAR (type I IFN receptor)-JAK (Janus kinase)-STAT (signal transducer and activator of transcription) signaling pathway, which are suppressed by accessory proteins, will be discussed. All cellular proteins interacted with paramyxovirus accessory proteins were summarized in Table 1. Major cell lines used in each reference were listed in Table 2.

**TABLE 1:** List of cellular proteins interacted with paramyxovirus accessory proteins.

Interaction partner	Viral protein (virus)	References
IKKα	V (MeV); C (BPIV3, MeV, NiV, and SeV)	Pfaller and Conzelmann, 2008; Yamaguchi et al., 2014
IRF7	V (MeV, NiV, and SeV)	Pfaller and Conzelmann, 2008; Kitagawa et al., 2011
TRAF6	V (HPIV2)	Kitagawa et al., 2013
RIG-I	V (MeV, NiV, PIV5, PPRV, RPV, and SeV)	Sanz Bernardo et al., 2017; Sánchez-Aparicio et al., 2018; Morita et al., 2020
MDA5	V (BeiPV, BPIV3, CDV, HPIV2, JPV, MeV, MuV, NiV, PIV5, PPRV, RPV, and SeV)	Andrejeva et al., 2004; Komatsu et al., 2007; Sakaguchi et al., 2011; Schaap-Nutt et al., 2011; Svitek et al., 2014; Audsley et al., 2016; Sanz Bernardo et al., 2017
UBXN1	V (NiV)	Uchida et al., 2018
PP1α/γ	V (MeV and NiV)	Davis et al., 2014
LGP2	V (HeV, HPIV2, MeV, MuV, NiV, PIV5, and PPRV)	Parisien et al., 2009; Sanz Bernardo et al., 2017
TBK1	V (HPIV2, MuV, and PIV5);	Lu et al., 2008
IKKε	V (HPIV2, MuV, and PIV5)	Lu et al., 2008
IRF3	P (PPRV); V (MeV, NDV, and SeV); C (HPIV1)	Irie et al., 2012; Li et al., 2021
IRF5	P (PPRV)	Li et al., 2021
IRF8	P (PPRV)	Li et al., 2021
p65	V (MeV)	Schuhmann et al., 2011
NLRP3	V (MeV and SeV); C (HPIV3)	Schuhmann et al., 2011; Komatsu et al., 2018; Shil et al., 2018
Caspase-1	V (HPIV2)	Ohta et al., 2018
IFNAR2	C (SeV)	Kitagawa et al., 2020
STAT1	P (MeV, NiV, PPRV, and RPV); V (CDV, HPIV4, MeV, MuV, NiV, PPRV, and RPV); W (NiV); C (RPV and SeV)	Garcin et al., 2002; Nishio et al., 2002; Takeuchi et al., 2003; Shaw et al., 2004; Nishio et al., 2005; Nanda and Baron, 2006; Caignard et al., 2007; Devaux et al., 2007, 2013; Svitek et al., 2014; Ma et al., 2015; Oda et al., 2015; Li P. et al., 2019; Keiffer et al., 2020; Nagano et al., 2020
STAT2	V (CDV, HPIV4, MeV, MuV, NiV, PIV5, and PPRV)	Nishio et al., 2002, 2005; Rodriguez et al., 2004; Ramachandran et al., 2008; Svitek et al., 2014; Nagano et al., 2020
RACK1	V (MuV)	Kubota et al., 2002
DDB	V (HPIV4 and PIV5)	Andrejeva et al., 2002a; Nishio et al., 2005; Precious et al., 2005a
TRIM25	V (MeV, NiV, PIV5, and SeV)	Sánchez-Aparicio et al., 2018
Tetherin	V (HPIV2, HPIV4, MuV, PIV5, and SV41)	Ohta et al., 2016, 2017a,2017b
p53	V (MeV)	Cruz et al., 2006
p73	V (MeV)	Cruz et al., 2006
TXNL1	V (NDV)	Chu et al., 2018
CacyBP/SIP	V (NDV)	Wang et al., 2018
MSI1	V (NDV)	Yang et al., 2021
FTH1	V (HPIV2)	Ohta et al., 2021
SNAP29	P (HPIV3)	Ding et al., 2014

**TABLE 2:** Major cell lines used in references.

References	Cell lines
Shaw et al., 2005	HEK293T
Melchjorsen et al., 2005	U937 and RAW264.7
Pfaller and Conzelmann, 2008	Hela
Kitagawa et al., 2011	HEK293T
Kitagawa et al., 2013	HEK293T
Yamaguchi et al., 2014	HEK293T
Sanz Bernardo et al., 2017	Vero cells expressing the human form of the morbillivirus receptor/Vero cells expressing canine SLAM
Morita et al., 2020	RAW264.7
Sánchez-Aparicio et al., 2018	Hela, HEK293T, and A549
Andrejeva et al., 2004;	Vero, HEK293T, and Hela
Sakaguchi et al., 2011	HEK293T
Svitek et al., 2014	Huh7
Audsley et al., 2016	Vero
Uchida et al., 2018	HEK293T
Schaap-Nutt et al., 2011	LLC-MK2
Davis et al., 2014	HEK293T
Parisien et al., 2009	HEK293T
Rodriguez and Horvath, 2014	HEK293T
Sun et al., 2019	Hela and HEK293T
Kiyotani et al., 2007	C57BL/6J mice
Irie et al., 2012	C57BL/6 mice and ICR mice
Boonyaratanakornkit et al., 2011	A549
Li et al., 2021	HEK293T and primary goat fibroblasts
Lu et al., 2008	HEK293T
Yokota et al., 2008	U937, THP-1
Schuhmann et al., 2011	HEK293T
Komatsu et al., 2018	HEK293T
Ohta et al., 2018	HEK293T, THP-1
Shil et al., 2018	THP-1
Kitagawa et al., 2020	HEK293T
Takeuchi et al., 2003	Hela
Caignard et al., 2007	HEK293T
Ramachandran et al., 2008	HEK293T and 2fTGH
Nagano et al., 2020	None
Devaux et al., 2007,	Hela, vero cells expressing human SLAM
Devaux et al., 2013	U3A
Kubota et al., 2005	Vero and CV1
Kubota et al., 2002	Human amnion cells (FL) and human lymphoblastoid cells of B-cell origin (Akata cells)
Nishio et al., 2002	MEF and BSR-T7
Yokosawa et al., 2002	Human amnion cells (FL) and HEK293T
Shaw et al., 2004	Hela, HEK293T, and A549
Keiffer et al., 2020	HEK293T
Rodriguez et al., 2004	2fTGH, HEK293T, U3A, and U6A
Rodriguez et al., 2003	2fTGH and HEK293T
Andrejeva et al., 2002a	2fTGH, HEK293T, and Hela
Precious et al., 2005a	HEK293T
Parisien et al., 2001	HEK293T
Andrejeva et al., 2002b	2fTGH
Precious et al., 2005b	MDBK, RK-13, Vero, MRC-5, Hep2, HDF, HD-MY-Z, and NBL-6
Nishio et al., 2005	2fTGH and Hela
Huang et al., 2003	Vero, DF-1, and 2fTGH
Qiu et al., 2016	A549, Vero, and DF-1
Wang et al., 2019	Chicken embryonic fibroblast cells (CEFs)
Ma et al., 2015	Cos7 and HEK293T
Li P. et al., 2019	HEK293T and Hela
Garcin et al., 2002	MEF and BSR-T7
Oda et al., 2015	HEK293T
Gotoh et al., 2003	Hela and U3A
Nanda and Baron, 2006	A549 and Vero
Ohta et al., 2016	Hela
Ohta et al., 2017b	HEK293T
Ohta et al., 2017a	Hela
Takeuchi et al., 2008	Hela, Vero, and U118
McAllister et al., 2010	Hela and Vero
Pfaller et al., 2014	Hela
Okonski and Samuel, 2013	Hela and Vero
Li Z. et al., 2019	Hela
Ringel et al., 2019	Vero76
Zhang et al., 2013	Hela
Hu et al., 2018	Hela
Yang et al., 2018	Caprine endometrial epithelial cells (EECs)
Ding et al., 2014	Hela and MK2
Li Y. et al., 2019	Hela
Sun et al., 2004	Hela and U3A
Rosas-Murrieta et al., 2011	C33
Cruz et al., 2006	HEK293T and 2fTGH
Chu et al., 2018	DF-1
Wang et al., 2018	DF-1
Yang et al., 2021	DF-1
Ohta et al., 2021	Hela
Bhattacharjee et al., 2019	Hela

### Toll-Like Receptors Signaling Pathway

Toll-like receptors are type I transmembrane proteins that play pivotal roles in recognizing invading pathogens. TLRs share homologous domain organization with N-terminal leucine-rich repeats (LRRs) followed by a single transmembrane region and a cytoplasmic TIR domain (Fitzgerald and Kagan, 2020). Viral infection activates the TLRs signaling pathway, then the MyD88, TRIF, Mal, and/or TRAM are recruited, subsequently activating IRF3 and IRF7 to promote the expression of IFN-β further.

**FIGURE 2:**
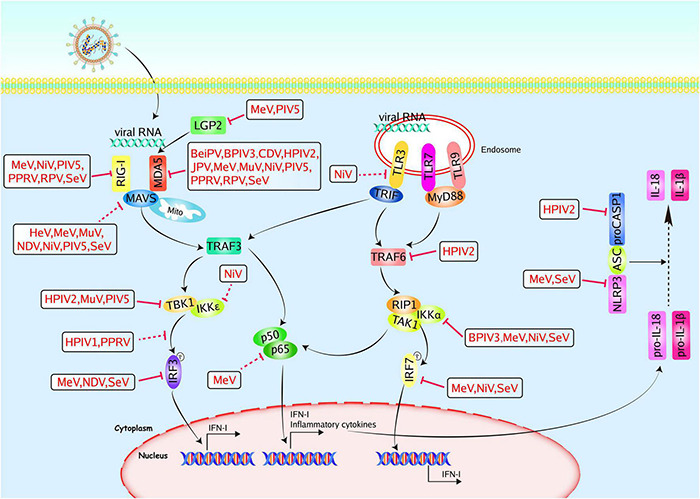
Paramyxoviruses accessory proteins-mediated evasion of the PRR-mediated antiviral innate immunity. PRRs, such as TLRs, and RLRs, could recognize pathogen-associated molecular patterns. TLR3/7/9 locate at endosomes. They sense viral RNA and signal through TRIF or MyD88 to activate IRFs and NF-κB. RIG-I and MDA5 recognize viral RNA and signal through MAVS to activate IRF3 and NF-κB. The NLRP3-mediated inflammasome pathway operates as a platform for the maturation of IL-1β and IL-18. Paramyxoviruses accessory proteins can hijack multiple steps in the PRR-mediated antiviral innate immunity. The V proteins of MeV, NiV, PIV5, PPRV, RPV, and SeV could inhibit RIG-I, and the V proteins of BeiPV, BPIV3, CDV, HPIV2, JPV, MeV, MuV, NiV, PIV5, PPRV, RPV, and SeV could inhibit MDA5. The V proteins of MeV and PIV5 could inhibit LGP2, and the V proteins of HeV, MeV, MuV, NDV, NiV, PIV5, and SeV could inhibit MAVS. The V proteins of HPIV2, MuV, and PIV5 could inhibit TBK1, the V and W proteins of NiV could inhibit IKKε. The PPRV P protein could inhibit TBK1-IRF3 interaction and the V proteins of MeV, NDV, and SeV could inhibit IRF3. The HPIV1 C protein could prevent the phosphorylation of IRF3. The P, V, and C proteins of MeV could inhibit p65. The NiV W protein could inhibit TLR3 and the HPIV2 V protein could inhibit TRAF6 and caspase1, respectively. The C proteins of BPIV3, MeV, NiV, and SeV could inhibit IKKα, the V proteins of MeV, NiV, and SeV could inhibit IRF7. The V proteins of MeV and SeV could inhibit NLRP3. Red solid lines indicate confirmed interactions between adaptors and accessory proteins, and red dashed lines indicate uncertain interactions or unknown underlying mechanisms; P, phosphate.

TLR3, TLR7, TLR8, and TLR9 have been shown to recognize paramyxoviruses and induce IFN-I (Melchjorsen et al., 2005; Kitagawa et al., 2011, 2013; Cheng et al., 2014). Several accessory proteins have been shown to counteract TLRs-mediated signaling (Figure 2). A previous study demonstrated that TLR3 could colocalize with dsRNA produced by NDV replication in host cells and initiate the innate proinflammatory responses (Cheng et al., 2014). NiV W protein exhibits strong inhibition of TLR3 activated by extracellular dsRNA, while V protein had no effect (Shaw et al., 2005). SeV was recognized by ssRNA receptors TLR7 and TLR8 in myeloid cells (Melchjorsen et al., 2005). In contrast, TLR7 and TLR9 can induce a burst quantities of IFN-I in plasmacytoid dendritic cells upon virus infection. Recognition of PAMPs by TLR7/9 results in the recruitment of MyD88 and IL-1 receptor-associated kinase 4 (IRAK4), which then bind to IκB kinase α (IKKα), TRAF6, TRAF3, and IRAK1. Finally, IRF7 is phosphorylated by IKKα and IRAK1, thus activating transcription and production of IFN-I (Blasius and Beutler, 2010). Some paramyxovirus accessory proteins can suppress the TLR7- and TLR9-dependent pathways. The C-terminal domain of MeV V protein inhibits IKKα-dependent induction of IFN-α through interacting with IKKα and IRF7, thus impairing the production of IFN-α (Pfaller and Conzelmann, 2008). Further studies found that V-IRF7 interaction is common in paramyxoviruses, including HPIV2, SeV, MeV, and NiV. Mechanistically, a tryptophan-rich motif in the C-terminal domain, the key for IRF7 binding, is critical for suppressing the TLR7/9-dependent pathway (Kitagawa et al., 2011). Interestingly, HPIV2 V protein interacts with not only IRF7 but also MyD88, IKKα, or TRAF6. Furthermore, the knockout of TRAF6 disrupts the interaction between V protein and MyD88, IRF7, or IKKα, which suggests that TRAF6 mediates interactions. Ultimately, V protein was proved to inhibit lysine 63 (K63)-linked polyubiquitination of IRF7 mediated by TRAF6, consequently preventing TLR7/9-dependent IFN production (Kitagawa et al., 2013). Apart from V protein discussed above, the C protein of SeV can bind to IKKα and inhibit the phosphorylation of IRF7, leading to the inhibition of TLR7/9 signaling. However, compared to full size C protein (aa 1–204), Y1 (aa 24–204), and Y2 (aa 30–204) could bind to IKKα and exhibit less ability to suppress TLR7/9 signaling, indicating that N-terminal of C protein is not required for interaction with IKKα. Similarly, the TLR7/9-mediated IFN induction is inhibited by the C proteins of other paramyxoviruses, including bovine parainfluenza virus type 3 (BPIV3), MeV and NiV (Yamaguchi et al., 2014).

### (RIG-I)-Like Receptor Signaling Pathway

All members of the RLRs family share similar structures with a central helicase domain and a C-terminal domain. Both RIG-I and MDA5 have two N-terminal caspase activation and recruitment domains (CARDs), which mediate signal transduction via binding to MAVS (Takeuchi and Akira, 2010; Carty et al., 2021). Owing to lacking the CARD domain, LGP2 is commonly believed to regulate RIG-I and MDA5. RLRs bind to MAVS upon virus infection and activate TBK1 and IκB kinase ε (IKKε), leading to the induction of IRF3 and IRF7, which together with the transcription factor nuclear factor kappa-light-chain-enhancer of activated B cells (NF-κB) promote the expression of IFN-I, and large quantities of cytokines participated in antiviral innate immunity (Rehwinkel and Gack, 2020). Currently, significant progress has been made for the interaction between paramyxoviruses accessory proteins and the RLR signaling pathway (Figure 2).

#### RIG-I, MDA5, and LGP2

The V proteins from PPRV and Rinderpest virus (RPV) interacts with both RIG-I and MDA5. While the interaction between V and RIG-I is weaker than the interaction with MDA5. The V protein still exhibits the suppression of IFN production mediated by RIG-I signaling, though only to a limited degree (Sanz Bernardo et al., 2017). Recently, SeV V protein was demonstrated to inhibit TRIM25-mediated ubiquitination of RIG-I. Meanwhile, SeV V also suppresses the CARD-dependent interaction between RIG-I and MAVS, thus preventing the iNOS activation and NO production (Morita et al., 2020). Sánchez-Aparicio et al. (2018) found that V proteins of some paramyxoviruses (including MeV, SeV, NiV, and PIV5) could bind to the CARD domain of RIG-I and prevent the downstream RIG-I-mediated signaling.

MDA5-mediated production of IFN is repressed by V proteins of PIV5 (formerly known as simian virus type 5, SV5), Beilong virus (BeiPV), J virus (JPV), BPIV3, HPIV2, MuV, SeV, CDV, PPRV, NiV, and HeV. Furthermore, the cysteine-rich C terminus of PIV5 V alone is sufficient to interact with MDA5 and suppresses the IFN-β promoter (Andrejeva et al., 2004; Komatsu et al., 2007; Sakaguchi et al., 2011; Svitek et al., 2014; Audsley et al., 2016; Sanz Bernardo et al., 2017). Except for direct binding to MDA5, NiV V protein can assist UBX domain-containing protein 1 (UBXN1) to interact with MAVS competitively, consequently preventing the MDA5-mediated antiviral innate immune responses. The UBXN1 is identified as a host protein that interacts with the zinc-finger motif of NiV V protein. Binding to NiV V leads to the stabilization of UBXN1 and promotes the interaction between UBXN1 and MDA5, suppressing MDA5-dependent IFN production (Uchida et al., 2018). Further studies screened 20 mutant V proteins of HPIV2 to identify the key residues involved in MDA5 binding. The region comprised of 175–180 is essential for binding to MDA5 (Schaap-Nutt et al., 2011). Phosphatase PP1, such as PP1α and PP1γ, are key regulators of RIG-I and MDA5 activation. NiV and MeV V proteins directly interact with PP1α/γ, resulting in inhibition of MDA5-mediated production of IFN (Davis et al., 2014).

As a result of lacking CARD domain, LGP2 is not related to the direct activation of downstream signaling. The exact role of LGP2 in antiviral signaling remains unresolved, partly due to the controversial effects of LGP2 during RLR-mediated signaling. In some cases, the RLR-mediated IFN induction is reduced in the cells overexpressed with LGP2 protein. In contrast, in response to various RNA virus infections, the production of IFN-β is dramatically decreased in mice lacking LGP2, suggesting a positive role in RLR-dependent signaling (Bruns and Horvath, 2015). Consistent with MDA5, LGP2 is also targeted by V proteins of PIV5, HPIV2, MuV, SeV, NiV, and HeV. These V proteins bind to the C-terminal domain of LGP2 and interrupt the ATP hydrolysis mediated by LGP2. However, the function of these interactions needed further investigation (Parisien et al., 2009). Besides, PPRV V protein was also reported to interact with LGP2. This interaction has little effect on the inhibition of RIG-I signaling mediated by V protein. Sanz Bernardo et al. (2017) speculated that the low-affinity interaction with LGP2 compared to MDA5 is related to the activity.

Intriguingly, IFN-β luciferase activity is enhanced in low ectopic expression of LGP2 using a reporter gene assay. In contrast, high ectopic expression of LGP2 suppresses IFN-β luciferase activity. However, the underlying mechanisms need further studies. MeV or PIV5 V protein can antagonize MDA5 signaling by interacting with LGP2, thus preventing LGP2-mediated MDA5 enhancement. Meanwhile, LGP2 is demonstrated to interfere with RIG-I signaling, and the V-LGP2 interaction has little effect on RIG-I signal transduction (Rodriguez and Horvath, 2014).

#### Mitochondrial Antiviral-Signaling Protein

Mitochondrial antiviral-signaling protein (MAVS), also known as IFN-β promoter stimulator 1 (IPS-1), CARD-adaptor-inducing IFN-β (CARDIF), or virus-induced signaling adaptor (VISA), is the key molecule in the activation of IFN-I production by interacting with RIG-I and MDA5. MAVS comprises an N-terminal CARD, a proline-rich region (PRR), and a C-terminal transmembrane (TM) domain. The MAVS CARD binds to RLRs through CARD-CARD interaction, and MAVS PRR interacts with the TRAF family to further enhance downstream signal transduction (Ren Z. et al., 2020). A recent study showed that NDV V protein could recruit E3 ubiquitin ligase RNF5 to degrade MAVS through Lys 362 and Lys 461 ubiquitin. Furthermore, V proteins from other paramyxoviruses, such as SeV, MeV, HeV, MuV, NiV, and PIV5, were used to detect the ability to degrade MAVS. These proteins can degrade MAVS and suppress the IFN-β promoter activity (Sun et al., 2019).

#### IFN Regulatory Factor 3

IFN regulatory factor 3 is activated by TBK1- and IKKε-mediated phosphorylation. Dimerized IRF3 translocates to the nucleus, resulting in the activation of IFN-I and downstream genes production (Thompson et al., 2011). Mice lacking IFN signaling exhibit an early clearance of rSeV mutants (deficient in V expression), while the early clearance does not occur in mice lacking IRF3. These results indicate that SeV V protein antagonizes IRF3 dependent antiviral innate immune responses but is independent of IFN (Kiyotani et al., 2007). Co-immunoprecipitation assays showed that SeV V physically binds to IRF3 and reduces the IRF3 activation. Except for SeV V protein, the V proteins from MeV and NDV interact with IRF3, while MuV and NiV V proteins do not (Irie et al., 2012). Another group also demonstrated that NiV’s V and W proteins block the promoter activation in response to IKKε, and W protein reduced the phosphorylated IRF3 to prevent IFN production (Shaw et al., 2005). Consistent with NiV W, the C protein of HPIV1 is an important regulator of the antiviral innate immune signaling pathway, preventing the phosphorylation of IRF3. An F170S substitution aborted this inhibition in C protein (Boonyaratanakornkit et al., 2011). Recently, the P protein of PPRV was reported to bind to the IRF association domain of IRF3, which results in disassociation of TBK1-IRF3 interaction, leading to the decreased IRF3 phosphorylation and blocks IRF3-mediated IFN production. Furthermore, PPRV P protein also interacts with IRF5 and IRF8 with an unknown mechanism (Li et al., 2021). Besides, the V proteins from MuV, HPIV2, and PIV5 can suppress the IRF3-mediated gene induction by inhibiting the import of IRF3 to the nucleus. Further studies found that phosphorylation of IRF3 was inhibited in the presence of V. The underlying mechanism is that these V proteins are authentic substrates for IKKε/TBK1. They can compete out IRF3 to counteract antiviral innate immune responses (Lu et al., 2008).

#### NF-κB

When inducing stimuli to trigger IKK activation, IκB is phosphorylated, ubiquitinated, and degraded, leading to the releasing of NF-κB. The mammalian NF-κB family consists of p65 (RelA), p50, p52, c-Rel, and RelB. The major form of NF-κB is a heterodimer comprised of p65 and p50 subunits (Hayden and Ghosh, 2008). Released NF-κB dimers further translocate to the nucleus and induce the transcription of target genes. Ubiquitin-modifying enzyme A20, a negative host regulator of NF-κB, is upregulated in MeV-infected monocytic cells. And MeV P protein is sufficient to induce A20, consequently leading to the inhibition of NF-κB signaling pathway-mediated IFN production (Yokota et al., 2008). All products encoded by the MeV P gene (P, V, and C protein) can suppress the NF-κB signaling pathway. V protein exhibits the most efficiently inhibitory ability and targets the subunit p65 but not p50. Ectopic expression of V protein binds to p65 and abolishes nuclear translocation of p65, thus resulting in the suppression of NF-κB-mediated antiviral innate immune responses (Schuhmann et al., 2011).

### NLR Signaling Pathway

Besides TLRs and RLRs, NLRs are another subfamily of PRRs, playing key roles in host defense against paramyxoviruses. To date, more than 20 NLRs are identified in humans, and they are homologous in structure, and NLRs contain N-terminal effector domain, a central NOD domain, and C-terminal LRRs (Pei and Dorhoi, 2021). Among NLRs, NLRP3 (NOD-, LRR-, and pyrin domain-containing protein 3) is well-characterized. The NLRP3 inflammasome, comprised of NLRP3, apoptosis-associated speck-like protein (ASC), and caspase-1, can be activated by PAMPs, including viral RNA and proteins, leading to the induction of IL-1β and IL-18, thus inducing antiviral innate immune responses (Zhao and Zhao, 2020; Zheng, 2021) (Figure 2). MeV V protein interacts with NLRP3 and suppresses the NLRP3 inflammasome-mediated IL-1β secretion (Schuhmann et al., 2011). THP1 macrophages lacking NLRP3 inhibited the rSeV (deficient in V expression) – induced IL-1β secretion. SeV V binds to NLRP3 to inhibit the self-oligomerization of NLRP3 and suppresses inflammasome-dependent IL-1β secretion. Moreover, the inhibition of NLRP3 activation is shared by paramyxoviruses, including NiV, HPIV2, and SeV (Komatsu et al., 2018). The V protein of HPIV2 also binds to the C-terminal region of caspase-1 and represses the maturation of IL-1β mediated by caspase-1 (Ohta et al., 2018). Apart from V protein, the HPIV3 C protein interacts with NLRP3 and promotes proteasomal degradation of NLRP3, which results in the blockade of inflammasome activation (Shil et al., 2018).

### Type I IFN Receptor-Janus Kinase-Signal Transducer and Activator of Transcription Signaling Pathway and Its Downstream IFN-Stimulated Genes

#### Type I IFN Receptor-Janus Kinase-Signal Transducer and Activator of Transcription Signaling Pathway

**FIGURE 3:**
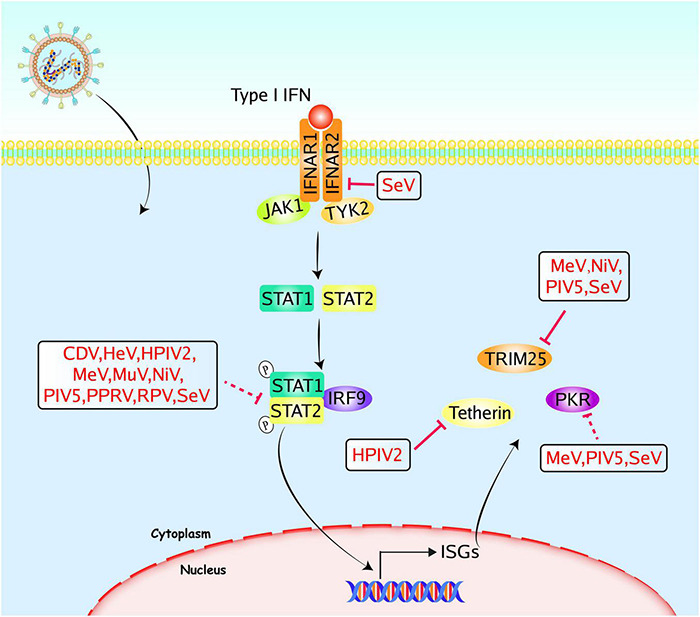
Paramyxoviruses accessory proteins-mediated evasion of the IFNAR-JAK-STAT signaling pathway. Following the binding of cytokines to the specific receptors, STATs are activated by members of the JAK family. Then they dimerize and translocate to the nucleus and regulate the expression of target genes, including ISGs. Accessory proteins from paramyxoviruses interact with adaptors to block the signal transduction, and some accessory proteins directly target ISGs to evade the innate immune responses. The V proteins of CDV, HeV, HPIV2, MeV, MuV, NiV, PIV5, PPRV, RPV, and SeV could inhibit STAT1/STAT2 mediated signaling pathways. The SeV C protein could inhibit IFNAR2, and the V proteins of MeV, NiV, PIV5, and SeV could inhibit TRIM25. The HPIV2 V protein could inhibit tetherin, the C proteins of MeV or SeV, and the MeV V protein could inhibit PKR. The PIV5 P and V proteins could inhibit the activation of PKR. Red solid lines indicate confirmed interactions between adaptors and accessory proteins, and red dashed lines indicate uncertain interactions or unknown underlying mechanisms; P, phosphate.

There are three major components in IFNAR-JAK-STAT signaling pathway: the cell receptor, JAK proteins, and STAT proteins. Following virus detection and IFN production, IFN binds to the IFNAR, activates JAK1 and tyrosine kinase 2 (TYK2), and recruits STAT proteins. Activation of the JAK-STAT pathway leads to the induction of ISGs, thus suppressing the virus replication (Ivashkiv and Donlin, 2014; Schneider et al., 2014). Many accessory proteins of paramyxoviruses can interfere with the IFNAR-JAK-STAT signaling pathway to evade the antiviral innate immune responses (Figure 3).

##### IFNAR2

IFN-I binds to the receptors composed of IFNAR1 and IFNAR2, and this heterodimeric receptor further phosphorylates JAK1 and TYK2 to activate the JAK-STAT signaling (Chen et al., 2017). SeV C protein interacts with STAT1 and suppresses the phosphorylation of STAT1 and STAT2 activated by IFN-α. However, the SeV C mutant, which cannot bind to STAT1, also retained the ability to interfere with IFN-I production. Through co-immunoprecipitation assay, SeV C was proved to interact with the cytoplasmic domain of IFNAR2. This interaction does not prevent STAT2 or JAK1 from binding to IFNAR2 but inhibits the tyrosine phosphorylation of JAK1 and TYK2 stimulated by IFN-I (Kitagawa et al., 2020).

##### STAT1 and STAT2

Janus Kinase family consists of four members, including JAK1, JAK2, JAK3, and TYK2. STAT family consists of seven members, include STAT1-4, STAT5a, STAT5b, and STAT6 (Raftery and Stevenson, 2017). Canonically, upon IFN-I activation, STAT1 and STAT2 are phosphorylated and heterodimerized. This heterodimer further recruits the IRF9 to form the trimeric complex, called IFN-stimulated gene factor 3 (ISGF3). Lastly, the ISGF3 translocates to the nucleus and binds to the IFN-stimulated response elements (ISREs) to initiate the transcription of various ISGs (Chen et al., 2017; Negishi et al., 2018). Many paramyxoviruses develop various strategies to inhibit the production of IFN-I mediated by STAT1 and/or STAT2.

#### Measles Virus

Measles virus is a highly contagious pathogen that is transmitted through the respiratory route and causes serious symptoms. The V protein of MeV can prevent IFN-induced nuclear accumulation of STAT1 and STAT2 by inhibiting STAT phosphorylation. MeV V was proved to be co-immunoprecipitated with STAT1 and STAT2. However, the interaction between STAT1 and V is greatly reduced in the absence of STAT2, indicating that STAT2 is required for this interaction. STAT2 is a primary target for MeV V protein (Takeuchi et al., 2003; Caignard et al., 2007; Ramachandran et al., 2008). However, Nagano et al. (2020) provided controversial evidence recently. They found that the different regions of V protein mediated the interactions with STAT1 or STAT2, and these interactions are independent of each other. Mechanistic studies clarified that the C-terminal region of V interacts with the STAT2-core region. This interaction competes with IRF9 for STAT2, leading to the disruption of ISGF3 formation and suppression of antiviral genes expression (Nagano et al., 2020). In addition to the V proteins discussed above, MeV P protein also interacts with the linker domain of STAT1 and suppresses the phosphorylation of STAT1. The tyrosine 110 is the key residue required to suppress STAT1 phosphorylation (Devaux et al., 2007, 2013).

#### Mumps Virus

Mumps virus infection usually causes fever and swelling of the parotid salivary glands. The exogenous expression of MuV V protein can block the IFN-β induced phosphorylation of STAT1 (Tyr 701) and STAT2 (Tyr 689) (Kubota et al., 2005). Mechanistic studies showed that MuV V interacts with the receptor for activated C kinase (RACK1), a key kinase that mediates the interaction between IFNAR and STAT1. By comparison, RACK1 binds to MuV V with a higher affinity than STAT1 does. Thus, MuV V interacts with RACK1, consequently suppressing IFNAR-STAT complex formation mediated by RACK1 (Kubota et al., 2002). The interactions were also observed between MuV V and STAT1/2. But the consequence of these interactions remains to be studied (Nishio et al., 2002). Moreover, MuV V also induces the ubiquitination and degradation of STAT1 (Yokosawa et al., 2002).

#### Nipah Virus and Hendra Virus

Nipah virus and HeV are two closely related members of the genus Henipavirus. Both of them cause severe respiratory illness and encephalitis. Accessory proteins of NiV also block the IFN-I mediated JAK-STAT signaling. Since three accessory proteins (P, V, and W) possess the common N-terminal domain, they all interact with STAT1 to block IFN production. The N-terminal residues 114 to 140 were found to interact with the STAT1 SH2 domain using a co-immunoprecipitation assay (Shaw et al., 2004; Keiffer et al., 2020). Interestingly, the NiV P and V mainly localize to the cytoplasm, whereas the NiV W predominantly localizes to the nucleus. Interestingly, all NiV accessory proteins colocalize with STAT1 and suppress STAT1 phosphorylation (Shaw et al., 2004). Among three accessory proteins, only V protein binds to STAT2. This interaction requires a large NiV V segment, including residues 100 to 300. Furthermore, deletion of residues 230–237 significantly decreased the interaction (Rodriguez et al., 2004). HeV is another member of Henipavirus, causing high morbidity and mortality in humans. Rodriguez et al. (2003) found that HeV V reduces IFN production by preventing STAT1/2 nuclear accumulation.

#### Parainfluenza Virus

Parainfluenza virus is a common respiratory pathogen that typically causes acute respiratory tract diseases in infants and immunocompromised adults. Both phosphorylated and non-phosphorylated forms of STAT1 could be reduced by PIV5 infection. Further studies found that bacterially expressed and purified PIV5 V protein results in the polyubiquitination and degradation of STAT1. Mechanistic studies proved that PIV5 V directly binds to the p127 subunit (DDB1) of the UV damage-specific DNA binding protein (DDB) and STAT2, but not to STAT1. PIV5 V is an adaptor molecule linking STAT1/STAT2 heterodimers to DDB1, which ultimately induces the ubiquitination of STAT1 (Andrejeva et al., 2002a; Precious et al., 2005a). Meanwhile, purified HPIV2 V protein induces the polyubiquitination and degradation of both STAT1 and STAT2 (Parisien et al., 2001; Andrejeva et al., 2002b; Precious et al., 2005b). Although HPIV4 V binds to STAT1, STAT2, and DDB1, it does not interfere with STAT phosphorylation and nuclear accumulation. As a result, HPIV4 cannot evade the antiviral innate immune responses mediated by IFN (Nishio et al., 2005).

#### Newcastle Disease Virus

Newcastle disease virus is a highly contagious pathogen among avian species resulting in enormous economic losses in poultry worldwide. NDV V is also considered a regulator of IFN-antagonism, promoting the degradation of phospho-STAT1 (Huang et al., 2003; Qiu et al., 2016). Recently, our group found that NDV V protein can activate the suppressor of cytokine signaling 3 (SOCS3) expression and suppress the IFN-I signaling (Wang et al., 2019).

#### Peste Des Petits Ruminants Virus

Peste des petits ruminants virus, which belongs to the genus Morbillivirus within the family *Paramyxoviridae*, causes fatal diseases in goats and sheep, leading to heavy economic losses. PPRV V directly binds to STAT1 and STAT2, which results in the suppression of IFN production. Ma et al. (2015) found that both the N-terminal and C-terminal domains of PPRV V inhibit the translocation of STATs. However, the N-terminal domain of V shows less ability to suppress IFN production. Further studies found that PPRV P also binds to STAT1 and suppresses the phosphorylation of STAT1 (Li P. et al., 2019).

#### Sendai Virus

Sendai virus, causing respiratory diseases in rodents, is considered a prototype of the family *Paramyxoviridae*. The unique C proteins are encoded by SeV P and V mRNAs. C proteins contain a nested set of proteins, including C (aa 1–204), Y1 (aa 24–204), Y2 (aa 30–204), and C′ (with an 11-aa addition to the N terminus of C). Garcin et al. (2002) demonstrated that all C proteins of SeV can form a complex with STAT1. However, only longer C proteins can target STAT1 for ubiquitination and degradation. Exogenous expression of Y proteins can not prevent IFN-mediated signaling. Recently, the C-terminal region of C protein (Y3; aa 99–204) was proved to form a STAT1:Y3 complex and reduce the dephosphorylation of STAT1, which results in the accumulation of phospho-STAT1. Finally, phospho-STAT1 and Y3 form the high-molecule-weight complexes, leading to the inhibition of IFN-γ signaling (Oda et al., 2015). The C protein was shown to inhibit phosphorylation of STAT2, and this process relies on the presence of STAT1, indicating that C protein acts on STAT2 through interacting with STAT1 (Gotoh et al., 2003; Oda et al., 2015).

#### Canine Distemper Virus

Canine distemper virus is highly contagious and known as a multi-host pathogen, causing a significant disease of wildlife. The CDV V binds to both STAT1 and STAT2. The wildtype V protein efficiently suppresses the STAT1 and STAT2 nuclear translocation, while the mutant V protein (lacking the ability to interact with STAT1 or STAT2) does not reduce the nuclear translocation of STATs (Svitek et al., 2014). These results suggest that CDV V protein plays an important role in the regulation of JAK-STAT signaling.

#### Rinderpest Virus

Rinderpest virus, which is related to the human virus MeV, has caused serious illness in cattle and has had a global impact. Nanda and Baron (2006) reported that RPV could block the phosphorylation and nuclear translocation of STAT1/2, thus suppressing the IFN-I production. All the RPV P, V, and C proteins could bind to STAT1, while none of these proteins bind to STAT2. Nevertheless, RPV V is the strongest inhibitor of IFN signaling. The RPV P and C proteins seem to weakly inhibit the IFN-mediated activation of STAT1 (Nanda and Baron, 2006).

### IFN-Stimulated Genes

Upon viral infection, IFN-I activates the JAK-STAT signaling, resulting in the formation of ISGF3. Activated ISGF3 translocates to the nucleus and drives the expression of ISGs. They play an important role in controlling viral infection. The tripartite motif-containing 25 (TRIM25), tetherin, and protein kinase R (PKR) will be discussed in this section.

#### TRIM25

Tripartite motif (TRIM) proteins are a large family of ubiquitin E3 ligase comprised around 70 members involved in multiple cellular processes. In recent years, many studies illustrated that TRIM proteins play important roles in antiviral host defenses. TRIM25, the first identified TRIM protein, can regulate RIG-I signaling by decorating the CARD domain within RIG-I with K63-linked polyubiquitin. Upon polyubiquitin, RIG-I is oligomerized and recruits the MAVS to induce antiviral gene expression (van Gent et al., 2018). Recently, Sánchez-Aparicio et al. (2018) reported that V proteins of paramyxoviruses (including MeV, NiV, SeV, and PIV5) could bind to the SPRY domain of TRIM25 and disrupt the TRIM25-mediated ubiquitination of RIG-I. These results suggest that various paramyxoviruses share the TRIM25-V interaction, and these interactions significantly suppress the TRIM25-mediated antiviral innate immune responses (Sánchez-Aparicio et al., 2018).

#### Tetherin

Tetherin, also known as BST2, CD317, or HM1.24, is a type II transmembrane protein that exhibits antiviral effects by inhibiting the release of enveloped viruses (Swiecki et al., 2013). Ohta et al. (2016, 2017b) found that HPIV2 V protein colocalizes with tetherin and binds to tetherin, which reduces cell surface tetherin. This reduction benefits the viral production of HPIV2 (Ohta et al., 2016, 2017b). It was reported that V proteins from other paramyxoviruses (including PIV5, MuV, HPIV4, and simian virus 41) were co-immunoprecipitated with tetherin. And infection of PIV5 or MuV reduced the expression of cell surface tetherin. However, they did not identify whether these V proteins sufficiently suppress tetherin expression (Ohta et al., 2017a).

#### Protein Kinase R

Viral infections can induce the halting of cellular protein expression, which is termed host shutoff. PKR, one of the well-characterized ISGs, is activated by dsRNA and subsequently phosphorylates the eukaryotic translation initiation factor eIF-2α, which modifies the host shutoff (Schoggins, 2019). Host shutoff could suppress the expression of cellular innate immune responses, benefiting the viral replication. Nevertheless, given the expression of viral proteins relies on host cells, viral protein synthesis could be affected by the host shutoff. Thus, some paramyxoviruses have evolved to replicate efficiently without inducing the host shutoff. Takeuchi et al. (2008) demonstrated that the cellular protein synthesis rate was suppressed by recombinant SeV lacking C protein (rSeVΔC) while remaining steady by the infection of wildtype SeV (wtSeV). Immunofluorescent staining experiments revealed the generation of a great amount of dsRNA with rSeVΔC but not wtSeV. However, the dsRNA generation was suppressed by exogenous C protein expression, indicating that SeV C limits dsRNA generation and suppresses PKR to maintain cellular protein synthesis (Takeuchi et al., 2008). Similarly, PIV5 P and V proteins could limit the activation of PKR through decreasing the synthesis of viral RNA and thus prevent the host shutoff (Gainey et al., 2008). McAllister et al. (2010) reported that PKR could enhance the production of IFN-β induced by recombinant MeV lacking V protein or C protein (rMeVΔV or rMeVΔC, respectively). Further studies proved that C protein plays a pivotal role in modulating PKR-mediated mitogen-activated protein kinase and NF-κB activation (McAllister et al., 2010). Pfaller et al. (2014) also found that rMeVΔC increased the amount of PKR activator RNA, thereby inducing antiviral innate immune responses.

## Accessory Proteins Inhibit the Apoptosis, Autophagy, and Stress Granules

### Apoptosis

Apoptosis is one of the major forms of programmed cell death, characterized by DNA fragmentation, chromatin condensation, and plasma membrane blebbing. It can be activated by two pathways, the intrinsic and extrinsic pathways (Zhou et al., 2017). The intrinsic apoptotic pathway is stimulated by a variety of intracellular signals including DNA damage, endoplasmic reticulum stress, oxidative stress, and cytokine deprivation. The extrinsic apoptotic pathway is elicited by extracellular stress stimulation via the activation of death receptors of the tumor necrosis factor receptors (TNFR) superfamily, leading to the activation of procaspase 8 to caspase 8 (Mehrbod et al., 2019).

**FIGURE 4:**
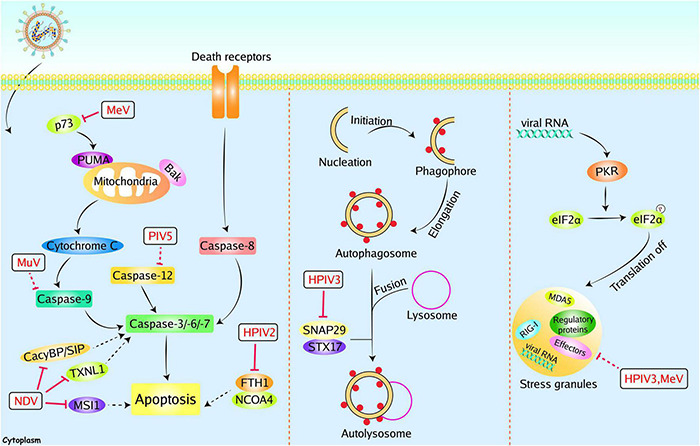
Paramyxoviruses accessory proteins-mediated evasion of apoptosis, autophagy, and SGs. Paramyxoviruses infection could induce other immune responses, including apoptosis, autophagy, and SGs to restrict viral replication. However, these immune responses can be manipulated by paramyxoviruses accessory proteins. The V proteins of NDV could inhibit apoptosis by interacting with CacyBP/SIP, TXNL1, or MSI1. The P protein of MeV could inhibit apoptosis with unknown mechanisms, and the MeV V protein could inhibit p73. The MuV V and PIV5 V protein could inhibit caspase 9 and caspase 12, respectively. The HPIV2 V protein could inhibit FTH1. The P protein of HPIV3 could inhibit autophagy through interacting with SNAP29. The P protein of HPIV3 and MeV C protein could inhibit stress granules (SG). Red solid lines indicate confirmed interactions between adaptors and accessory proteins, and red dashed lines indicate uncertain interactions or unknown underlying mechanisms; P, phosphate.

The initiation of apoptosis relies upon two categories of caspases including the initiator caspases (caspases 8 and 9) and the executioner caspases (caspases 3, 6, and 7) (Figure 4). During viral infection, apoptosis is a double-edged sword. On one hand, apoptosis can eliminate infected cells to interrupt viral proliferation. On the other hand, viruses can utilize apoptosis to release progeny viruses (Zhou et al., 2017). For instance, apoptosis assists NDV release and dissemination by killing cells (Li Y. et al., 2019). In contrast, apoptosis can eliminate Hepatitis B Virus (HBV) by aborting virus infection (Lin and Zhang, 2017). Consequently, viruses have evolved multiple strategies to regulate apoptosis to benefit replication. Sun et al. (2004) proved that recombinant PIV5 lacking the C-terminus of V protein (rPIV5ΔV_*C*_) triggers apoptosis by inducing endoplasmic reticulum stress. Using specific caspase inhibitors, they found that rPIV5ΔV_*C*_-induced apoptosis can arise in a caspase 12-dependent manner. Further studies found that rPIV5ΔV_*C*_ induced apoptosis can be prevented by exogenous expression of V protein, suggesting that V protein possesses anti-apoptotic effects (Sun et al., 2004). Besides, MuV V protein also exhibits an anti-apoptotic effect. Two V proteins inhibited apoptosis induced by IFN-α2b from variant MuV strains (Rosas-Murrieta et al., 2011). Recombinant MeV lacking V protein (rMeVΔV) induces more severe cell death, and exogenous expression of V protein alleviates the rMeVΔV-induced cell death. MeV V protein was proved to bind to both p53 and p73 DNA binding domains and specifically blocked the transcriptional activity of p73. These results may explain the anti-apoptotic effect of V protein (Cruz et al., 2006). Our group recently identified some proteins that interacted with NDV V protein by yeast’s two-hybrid screening system. For instance, NDV V protein interacts with and downregulates thioredoxin-like protein 1 (TXNL1) or CacyBP/SIP in DF-1 cells, inhibiting apoptosis and inducing viral replication (Chu et al., 2018; Wang et al., 2018). Another interacting protein, Musashi RNA binding protein 1 (MSI1), was identified by co-immunoprecipitation assay. Overexpression of MSI1 suppresses the extracellular virions without reducing the viral RNAs and proteins. It was proved that MSI1 inhibits NDV release by preventing cell apoptosis (Yang et al., 2021). Recently, the V protein of HPIV2 was found to modulate iron homeostasis and prevent apoptotic cell death, leading to efficient viral growth. The HPIV2 V protein was reported to interact with ferritin heavy chain 1 (FTH1) and prevent the interaction between FTH1 and nuclear receptor coactivator 4 (NCOA4), resulting in the inhibition of iron release to the cytoplasm. Finally, this iron homeostasis regulation assists host cells in avoiding apoptosis and promotes the viral production of HPIV2 (Ohta et al., 2021). In addition to the V proteins discussed above, the P protein of MeV exhibits anti-apoptotic function and enhances the viral production in Hela cells (Bhattacharjee et al., 2019).

### Autophagy

Macroautophagy/autophagy is a highly conserved degrative process that is required to maintain homeostasis. It can sequester cytoplasmic cargo into a cup-shaped double-membrane and deliver this cargo to lysosomes to form autolysosomes, allowing for the degradation of cargo (Figure 4). Upon viral infection, autophagy can be stimulated as an innate immune response to prevent infection. In contrast, autophagy has been demonstrated to degrade and dispose of invading pathogens. The autophagy processes are utilized by some viruses to benefit their own replication, including hepatitis C virus, classical swine fever virus, and influenza virus. Autophagosomes can provide a protective environment for these viruses, and viruses can take advantage of the energy and metabolites generated by autophagy (Deretic et al., 2013; Choi et al., 2018). NDV infection induces steady-state autophagy, and inhibition of autophagy reduces NDV replication (Sun et al., 2014). Infection of PPRV also induces autophagy and inhibits apoptosis, thus enhancing the replication of PPRV (Yang et al., 2018). These results indicate that autophagy is a double-edged sword during viral infection. HPIV3 infection induces the accumulation of autophagosomes and incomplete autophagy, which promotes extracellular viral production. And the P protein of HPIV3 is sufficient to trigger incomplete autophagy. A synaptosome-associated protein of 29 kDa (SNAP29) was found to bind to P protein by a yeast two-hybrid system and verified by a co-immunoprecipitation assay. Further studies proved that P suppresses autophagosome-lysosome fusion through blocking SNAP29 interaction with syntaxin17, which benefits the viral production (Ding et al., 2014).

### Stress Granules

Stress granules are cytoplasmic foci induced by cellular stress or viral infection. Under stressed-condition, the α subunit of eukaryotic initiation factor 2 (eIF2α) is inactivated by phosphorylation at serine 51. Phosphorylation of eIF2α blocks the global translation initiation, resulting in the recruitment of translation-stalled mRNAs by RNA-binding proteins to induce the formation of SGs (Wang et al., 2020) (Figure 4). SGs can function as novel antiviral innate immune responses. For instance, SGs-mediated translation arrest can block the viral protein synthesis (McCormick and Khaperskyy, 2017). Furthermore, RIG-I and MDA5 were found to localize in SGs, and RIG-I stimulates the production of IFN-I (Onomoto et al., 2012; Langereis et al., 2013). Given the antiviral effect of SGs, some paramyxoviruses evolved multiple strategies to interfere with SG formation. For instance, the C protein from MeV can block SG formation (Okonski and Samuel, 2013). SGs also play an inhibitory role in viral replication by sequestering viral mRNA. Thus, protecting viral mRNA from SGs is another effective strategy. Inclusion bodies (IBs), or viral replication factories, are composed of viral proteins and RNA aggregates. Members of the *Mononegavirales* can form IBs to promote their replication. Due to the aggregation of viral RNA and proteins, IBs are considered viral replication and transcription sites. Recently, HPIV3 and NiV infection have been proved to induce the formation of IBs (Li Z. et al., 2019; Ringel et al., 2019). Although SGs have an antiviral effect against HPIV3, this effect is independent of IFN induction. The P protein formed IBs with N protein to counteract the SGs formation (Zhang et al., 2013). Hu et al. (2018) found that mRNAs of HPIV3 trigger SGs formation in a PKR-dependent manner. Co-expression of N and P enhances the formation of IBs and effectively disrupts the formation of SGs. Through RNA-FISH assay, Hu et al. (2018) illustrated that IBs formation specifically shields viral RNAs, consequently leading to the blockade of HPIV3-induced SGs. Ultimately, inhibition of SGs formation results in the enhancement of HPIV3 replication (Hu et al., 2018).

## Conclusion and Future Perspectives

In summary, the interplay between paramyxoviruses and the host antiviral innate immunity is extremely intricate. Although the host cells developed various mechanisms to recognize and block viral replication, paramyxoviruses have evolved corresponding immune evasion strategies to counteract the host innate immunity. The accessory proteins are major modulators in this battle. These proteins efficiently antagonize both the recognition and IFN-mediated clearance of viruses. TLRs, RLRs, and STATs and are all targeted by accessory proteins. Besides combating the canonical IFN-I signaling, paramyxoviruses accessory proteins could exploit other antiviral innate immune responses (such as SGs, autophagy, and apoptosis) to benefit viral replication. Notably, substantial progress has been made in understanding the host antiviral components. Here, we concluded and discussed the currently known confrontation strategies employed by accessory proteins of paramyxoviruses. Most studies elucidated the mechanisms of paramyxoviruses evasion of PRR-mediated antiviral innate immunity. Nevertheless, there remain some blank spaces to be further investigated. For example, although autophagy plays a critical role in paramyxovirus infection, only HPIV3 P protein was proved to inhibit the formation of autolysosome. Other evasion strategies utilized by paramyxoviruses accessory proteins remained to be investigated. Besides, most of these studies were conducted in cell cultures. It is crucial and interesting to assess the evasion strategies utilized by paramyxoviruses accessory proteins *in vivo*. Further, the critical challenge is to utilize these findings for the development of novel antiviral drugs. In addition to the signaling pathways mentioned in this manuscript, it will be important to evaluate other immune responses, such as the DNA damage response (DDR). Ataxia telangiectasia-mutated (ATM) protein kinase, ataxia telangiectasia and Rad3-related (ATR), and DNA-dependent protein kinase (DNA-PK) are three primary mediators that regulate the DNA damage response (DDR), which is a self-protection mechanism for the cells. Besides, the DDR sensors, such as DNA-PK and meiotic recombination 11 homolog A (MRE11), also participate in facilitating the production of type I IFN (Zhu and Zheng, 2020). The DDR signaling pathway plays a critical role in virus infection, especially the DNA virus. Accordingly, HSV-1 has evolved evasion strategies to suppress DDR signaling pathway. ICP0 has been found to restrain the activation of ATM response (Su et al., 2016). Recently, Ren S. et al. (2020) found that the NDV replication and membrane fusion triggered ATM-mediated DNA double-strand break (DSB), which facilitates NDV replication. Thus, it will be necessary to investigate whether the paramyxoviruses accessory proteins could facilitate DDR signaling. The cyclic guanosine monophosphate-adenosine monophosphate (cGAMP) synthase (cGAS), playing an important role in antiviral innate immunity, has been identified as a universal cytoplasmic DNA sensor. Once activated by the DNA-containing pathogens, cGAS dimerizes and further oligomerizes to generate endogenous cGAMP, which serves as a second messenger. Next, the cGAMP binds to the ligand-binding domain of the stimulator of interferon genes (STING) and leads to the STING activation. Upon activation, STING translocates from the endoplasmic reticulum to the Golgi apparatus and triggers the type I IFN signaling pathway through recruiting TBK1 and IRF3 (Hopfner and Hornung, 2020; Zheng, 2021). However, little is known about the function of the cGAS/STING signaling pathway during RNA virus infection. Does the cGAS/STING signaling pathway contribute to the control of paramyxoviruses? Surprisingly, during the preparation of this manuscript, a recent study from Horvat’s group has shown that the NiV and MeV can induce the cGAS/STING axis, which appears to be involved in the control of paramyxoviruses by inducing the type I IFNs (Iampietro et al., 2021). These results lead us to question that whether paramyxoviruses accessory proteins could regulate the cGAS/STING axis. Further understanding of these questions will provide more mechanisms and details of paramyxoviruses infection.

## Author Contributions

CW, TW, LD, HC, and RH wrote the draft. HL, XW, SZ, RD, SX, JW, and ZY edited and revised the manuscript. All authors contributed to the article and approved the submitted version.

## Conflict of Interest

The authors declare that the research was conducted in the absence of any commercial or financial relationships that could be construed as a potential conflict of interest.

## Publisher’s Note

All claims expressed in this article are solely those of the authors and do not necessarily represent those of their affiliated organizations, or those of the publisher, the editors and the reviewers. Any product that may be evaluated in this article, or claim that may be made by its manufacturer, is not guaranteed or endorsed by the publisher.
